# A Gut-Ex-Vivo System to Study Gut Inflammation Associated to Inflammatory Bowel Disease (IBD)

**DOI:** 10.3390/biology10070605

**Published:** 2021-06-30

**Authors:** Mara Gagliardi, Romina Monzani, Nausicaa Clemente, Luca Fusaro, Valentina Saverio, Giovanna Grieco, Elżbieta Pańczyszyn, Nissan Yissachar, Francesca Boccafoschi, Marco Corazzari

**Affiliations:** 1 Department of Health Science, University of Piemonte Orientale, 28100 Novara, Italy; mara.gagliardi@uniupo.it (M.G.); romina.monzani@uniupo.it (R.M.); nausicaa.clemente@uniupo.it (N.C.); valentina.saverio@uniupo.it (V.S.); 20020550@studenti.uniupo.it (G.G.); elzbieta.panczyszyn@uniupo.it (E.P.); francesca.boccafoschi@uniupo.it (F.B.); 2 Center for Translational Research on Autoimmune and Allergic Disease (CAAD), University of Piemonte Orientale, 28100 Novara, Italy; 3 Interdisciplinary Research Center of Autoimmune Diseases (IRCAD), University of Piemonte Orientale, 28100 Novara, Italy; 4 Tissuegraft srl, 28100 Novara, Italy; luca.fusaro@uniupo.it; 5 The Mina and Everard Goodman Faculty of Life Sciences, Bar-Ilan Institute of Nanotechnology and Advanced Materials, Bar-Ilan University, Ramat-Gan 5290002, Israel; nissan.yissachar@biu.ac.il

**Keywords:** IBD, tissue culture, UPR, ex vivo organ, DNBS

## Abstract

**Simple Summary:**

Inflammatory Bowel Disease (IBD) is a complex and multifactorial systemic disease of the gastrointestinal tract, characterized by chronic inflammation, thus resulting in tissue damage and, occasionally, in cancer development. Although the precise origin is still elusive, it is widely considered a disease of modern society, caused by a complex interaction between environment, genetic, immune system, and gut microflora (microbiota). Potentially affected by all the above-mentioned variables, which interplay are highly heterogeneous, the disease appears to be patient-specific. The latter phenomenon, together with the uncertain origin, also contributes to the lack of optimal clinical treatment of these patients. Therefore, the development of appropriate models is crucial to push the research forward and to define new valuable therapeutic approaches. Although tissue biopsies and/or animal models represent the best models to study IBD onset, progression, and clinical interventions, they are both affected by limitations such as invasiveness, cost- and time-consuming, and ethical issues such as animal suffering. Here we propose a novel approach based on the cultivation of mouse tissues (colon) in an ex vivo microfluidic device (Gut-Ex-Vivo System, GEVS) to study IBD. We demonstrate that explanted mouse tissues cultivated in our GEVS can be appropriately stimulated to recapitulate the onset of the disease, in a time- and cost- effective manner.

**Abstract:**

Inflammatory bowel disease (IBD) is a complex, chronic, and dysregulated inflammatory condition which etiology is still largely unknown. Its prognosis and disease progression are highly variable and unpredictable. IBD comprises several heterogeneous inflammatory conditions ranging from Ulcerative Colitis (UC) to Crohn’s Disease (CD). Importantly, a definite, well-established, and effective clinical treatment for these pathologies is still lacking. The urgent need for treatment is further supported by the notion that patients affected by UC or CD are also at risk of developing cancer. Therefore, a deeper understanding of the molecular mechanisms at the basis of IBD development and progression is strictly required to design new and efficient therapeutic regimens. Although the development of animal models has undoubtedly facilitated the study of IBD, such in vivo approaches are often expensive and time-consuming. Here we propose an organ ex vivo culture (Gut-Ex-Vivo system, GEVS) based on colon from Balb/c mice cultivated in a dynamic condition, able to model the biochemical and morphological features of the mouse models exposed to DNBS (5–12 days), in 5 h. Indeed, upon DNBS exposure, we observed a dose-dependent: (i) up-regulation of the stress-related protein transglutaminase 2 (TG2); (ii) increased intestinal permeability associated with deregulated tight junction protein expression; (iii) increased expression of pro-inflammatory cytokines, such as TNFα, IFNγ, IL1β, IL6, IL17A, and IL15; (iv) down-regulation of the anti-inflammatory IL10; and (v) induction of Endoplasmic Reticulum stress (ER stress), all markers of IBD. Altogether, these data indicate that the proposed model can be efficiently used to study the pathogenesis of IBD, in a time- and cost-effective manner.

## 1. Introduction

Gut inflammation is associated with many different human pathologies such as Celiac Disease, cancer, obesity, diabetes, and IBD [1]. The latter condition consists of a not yet well-determined multifactor and heterogeneous pathology, ranging from Crohn’s Disease to Ulcerative Colitis [2]. Chronic inflammation associated with IBD, therefore, causes a dysfunction of the intestinal barrier permeability, altered morphology and tissue damage, and, occasionally, the development of cancer [3]. As part of the large chronic immune-mediated inflammatory diseases (IMIDs) including multiple sclerosis, rheumatoid arthritis, psoriasis, and others [4], it is considered a disease of modern society [5]. Although still elusive, the main components contributing to IBD onset and progression are thought to be the environment, genetic, microbiome, and immune system, which interplay are highly heterogeneous, giving rise to patient-specific disorders [6]. Potentially affected by all the above-mentioned variables, the current treatment options for IBD are far from optimal. Therefore, brand new perspectives and novel approaches to understand IBD and its treatment must be considered, investigated, and eventually applied.

Although patient-derived tissue biopsies might represent the “gold standard” mate-rial to study, this is the result of an invasive sampling which, usually, lacks an appropriate “healthy control”. Moreover, these tissues (a very low amount of material) can be maintained in culture for a very limited time. On the other hand, cell cultures (either 2D or 3D) can produce a large number of samples, with a system that can be easily manipulated (treated with different compounds and for a long time). However, data obtained by these systems are very limited and not representative of the whole tissue/organ. In this context, animal models represent the best option, whether an animal model is able to recapitulate the human condition. However, animals are expensive, experiments are time-consuming and their use is even more limited by ethical issues such as animal suffering. Here we propose an ex vivo organ culture (Gut-Ex-Vivo System, GEVS), to cultivate mice colon tissues to study gut inflammation associated with IBD.

## 2. Materials and Methods

### 2.1. Reagents and Materials

DNBS (2,4-Dinitrobenzenesulfonic acid hydrate; CAS Number: 698999-22-3), Thapsigargin (CAS Number: 67526-95-8; ≥98% (HPLC)), Cell Lytic buffer, protease inhibitors cocktail (PIC), nitrocellulose membrane, PBS (phosphate buffer solution), Tween20 (CAS Number: 9005-64-5), poly(dimethylsiloxane) (PDMS; Sylgard 184 Elastomer base), formaldehyde solution (CAS Number: 50-00-0), and Paraffin (CAS Number: 8002-74-2) were from Merck (Merck Life Science, Milan, Italy); hematoxylin (CAS Number: 517-28-2), eosin (CAS Number: 548-24-3) were from Bio-Optica; MTS, Cell- Titer 96^®^ AQueous Non-Radioactive Cell Proliferation Assay, 3-(4,5-dimethylthiazol-2-yl)-5-(3-carboxymethoxyphenyl)-2-(4-sulfophenyl)-2H-tetrazolium solution (CAS Number: 298-93-1) and AMV Reverse Transcriptase were from Promega (Promega Italia, Milan, Italy); IMDM (Iscove’s Modified Dulbecco’s Medium), KnockOut serum replacement, B-27, N-2 supplements, L-glutamine, non-essential amino acids (NEAA) and HEPES were from GIBCO; Dulbecco’s Modified Eagle’s Medium (DMEM), fetal bovine serum (FBS) and Penicillin/Streptomycin were from Euroclone (Euroclone, Milan, Italy); TripleXtractor was from GRiSP (Bio-Cell, Rome, Italy); non-fat dry milk was from Bio-Rad; anti-GrP78, anti-Calnexin, and anti-Tubulin were from Santa Cruz (DBA Italia, Segrate, Italy); anti-Calreticulin was from Abcam (Abcam, Cambridge, UK); anti-cleaved PARP, anti-ATF4, and anti-Actin were from Cell Signaling (Cell Signaling Technology, Denver, MA, USA); anti-TG2 was from NeoMarkers (Novus Biologicals, Centennial, CO, USA); horseradish peroxidase (HRP)-conjugated secondary antibodies were from Jackson (Jackson ImmunoResearch, Ely, UK); ECL plus was from Amersham (Amersham Biosciences, Amersham, UK); Q Path^®^ Coverquick 2000 was from VWR (VWR International, Milan, Italy).

### 2.2. Silicone-Based Device and Organ Culture

Colon from 13 days old Balb/c mice was resected and cultivated in a silicone-based Gut-Ex-Vivo System (GEVS), as previously described [7]. Briefly, the custom-fabricated fluidic chip consists of a silicone-based device divided into 6 parallel isolated chambers to allocate mouse colons (up to six). Tissues are cultivated with a nutrient supply perfusing the colon lumen in a dynamic configuration, by using a coordinated infusing-drying pump. While the outer medium circuit is static. During the experiment, the temperature of the device is maintained at 37°C using a lab hot plate, and a mixture of 5% CO_2_ and 95% O_2_ is provided to the device from a compressed gas cylinder.

Prior to dissection, input/output ports were flushed using a sterile culture medium and colon fragments were gently flushed in a sterile medium and fixed over the luminal input and output ports. After inserting each tissue fragment into the chamber, the device is placed on the heat-conducting adapter and sealed [7,8].

### 2.3. Colon Cultures and Treatments

Each colon was infused with serum-free tissue culture medium containing IMDM, supplemented with 20% KnockOut serum replacement (Gibco, Life Technologies Italia, Monza, Italy), 2% B-27 and 1% of N-2 supplements, 1% L-glutamine, 1% NEAA, 1% HEPES and stimulated with DNBS (0.5–1.5–2.5 mg/mL) or Thapsigargin (5.0 μg/mL).

The tissue culture medium was loaded into a 5 mL syringe (for short-term experiments, 5 h) and infused into the device input ports by a syringe pump (flow rate of 99 μL/h) [7].

### 2.4. Tissue Viability Assay

Tissue viability was evaluated as previously described [7], through MTS assay. Tissues were placed in a 48 well plate and cultured for 4 h with DMEM supplemented with 10% FBS), 2mM L-glutamine, 100 U/mL Penicillin, 0.1 mg/mL Streptomycin (Euroclone) and 3-(4,5-dimethylthiazol-2-yl)-5-(3-carboxymethoxyphenyl)-2-(4-sulfophenyl)-2H-tetrazolium solution following manufacturer’s instructions. The absorbance of 100 μL of medium solution from each sample was measured by UV-VIS spectrophotometry (Victor X4, PerkinElmer, PerkinElmer Italia Spa, Milan, Italy), at a wavelength of 490 nm. Values were proportional to cell viability.

### 2.5. Quantitative PCR (qPCR)

TripleXtractor reagent (Grisp, Bio-Cell, Rome, Italy) was used to isolate total RNA, as indicated by the supplier. The AMV Reverse Transcriptase kit was used to generate cDNA following the manufacturer’s instructions. Quantitative PCR reactions were performed by using a CFX96 thermocycler (Bio-Rad, Segrate, Italy). Primers sequences are reported in Supplementary Table S1, and were designed by using the online IDT PrimerQuest Tool software (IDT; https://eu.idtdna.com/Primerquest/Home/Index; accessed on 6 February 2019). The GAPDH mRNA level was used as an internal control, and comparative Ct method (ΔΔCt) was used for relative quantification of gene expression [9].

### 2.6. Western Blotting Analysis

Tissues were lysed using the Cell Lytic buffer supplemented with PIC, subjected to an SDS-PAGE, and electroblotted onto nitrocellulose membranes. Membranes were blocked by using 5% non-fat dry milk in PBS plus 0.1% Tween20 (1 h) and incubated with indicated primary antibodies in blocking solution, overnight at 4 °C. Primary antibodies were: anti-GrP78 1:500, anti-Calnexin 1:500, anti-Calreticulin 1:500, anti-ATF4 1:500, anti-PARPcl 1:500, anti-Tubulin 1:10,000, and anti-αActin 1:2000. Detection was achieved using HRP-conjugated secondary antibodies (1:5000) and visualized with ECL plus. Images were acquired by using a ChemiDoc™ Touch Imaging System (Bio-Rad, Segrate, Italy) and analyzed by Image Lab software (Bio-Rad, Segrate, Italy), as previously described [10]; densitometry was performed by using the Image Lab software (Bio-Rad, Segrate, Italy).

### 2.7. ELISA

IFNγ, IL17A, and IL15 were measured in colon tissue lysates by using the mouse IFNγ Quantikine ELISA Kit, the mouse IL-17 Quantikine ELISA Kit, or the mouse IL-15 DuoSet ELISA (Bio-Techne, MN, USA), as recommended by the supplier. ODs were evaluated by a SPARK Multimode Microplate Reader (Tecan Group Ltd., Männedorf, Switzerland). Values were normalized to total protein concentration evaluated by Bradford analysis.

### 2.8. Hematoxylin/Eosin Staining

After being recovered from the GEVS, tissues were fixed in a 4% formaldehyde solution, dehydrated and embedded in paraffin. Following samples were then cut into sections of 5 μm, rehydrated and soaked in hematoxylin for 3 min, and in eosin solution (0.05% eosin, Sigma-Aldrich, in distilled water and acetic acid) for 6 min. Finally, samples were dehydrated, mounted with Q Path^®^ Coverquick 2000 (VWR International, Milan, Italy) and analyzed by an optic microscope (Leica DM2500, Leica, Wetzlar, Germany). Pictures have been acquired through a Leica DFC7000T camera (Leica Biosystems, Milan, Italy) and analyzed with Leica Application Suite X software (Leica Biosystems, Milan, Italy). Image analysis (thickness) was performed by using ImageJ software.

### 2.9. TUNEL

Apoptosis in tissue sections was evaluated by the ApopTag^®^ plus Peroxidase in situ Apoptosis detection kit (Merck Life Science, Milano, Italy). Briefly, paraffin-embedded samples were cut in 5 µm sections, then rehydrated and submerged into a 20 µg/mL proteinase K solution for 15 min. Subsequently, samples were placed in 3% H_2_O_2_ for 5 min and washed with ultrapure water. Sections were then immersed in Equilibration Buffer for 10 s, and incubated in a Terminal Deoxynucleotidyl Transferase (TDT) solution for 1 h at 37°C. Afterwards, samples were immersed in Stop/Wash solution for 10 min, and incubated with Anti-Digoxigenin Peroxidase conjugated antibody for 30 min at room temperature. Then, samples were washed with PBS and incubated with the Peroxidase substrate solution for 5 min. Finally, sections were dehydrated, mounted with Coverquick 2000 mounting medium (VWR International, Milan, Italy), acquired with Pannoramic MIDI (3DHISTECH Ltd., Budapest, Hungary), and analyzed with Pannoramic Viewer software (3DHISTECH Ltd., Budapest, Hungary).

### 2.10. Statistical Analysis

Experiments were performed in triplicate and repeated at least three times, and statistical analysis was performed using GraphPad software (GraphPad Software; GraphPad Prism 6). Student’s t test or ANOVA was used to determine statistical significance.

A *p*-value of equal to or less than 0.05 was considered significant. mRNA expression levels were represented as ‘fold change over control’, r.l. relative levels. Histograms represent mean ± SD; **** *p* < 0.0001; *** *p* < 0.001; ** *p* < 0.01; * *p* < 0.05; ns non-significant.

## 3. Results

### 3.1. Ex Vivo Culturing and Viability of Colon Tissues—GEVS

**Figure 1:**
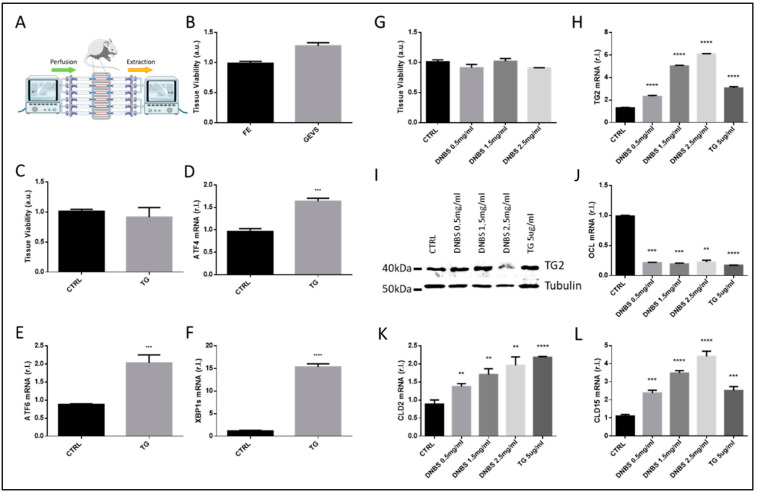
GEVS configuration and DNBS-stimulated tissue stress and altered permeability. (**A**) GEVS schematic representation in which the configuration of chambers is highlighted. (**B**) Tissue viability of colon cultivated 5 h in the gut-ex-vivo system (GEVS) or freshly explanted (**E**,**F**). The colon was cultivated 5 h in the GEVS and untreated (CTRL) or treated with Thapsigargin (TG), and tissue viability was evaluated by MTT Assay (**C**), or total RNA was isolated and ATF4 (**D**), ATF6 (**E**) or spliced XBP1 (XBP1s, (**F**)) expression levels were evaluated by qPCR. Colon was cultivated 5 h in a GEVS and untreated (CTRL) or treated with the indicated concentrations of DNBS, or Thapsigargin (TG), tissue viability was evaluated by MTT assay (**G**) and cellular stress was evaluated by measuring the expression of TG2, by qPCR (**H**) and western blotting analysis (**I**). Tissue permeability was evaluated by measuring the mRNA levels of Tight Junction (TJ) components Occludin (OCL, (**J**)), Claudin-2 (CLD2, (**K**)), or Claudin-15 (CLD15, (**L**)), by qPCR. Each panel is representative of experiments performed in triplicate. Histograms represent mean ± SD of triplicate sample; **** *p* < 0.0001; *** *p* < 0.001; ** *p* < 0.01.

To generate a colon ex vivo culturing, we used the previously described GEVS [7,8]. To this aim, colon from 13-days-old Balb/c mice was surgically resected, flushed in sterile conditions, and inserted into a silicon-based device. Each colon lumen (in each individual chamber, up to six) was perfused with a complete medium with additional treatments, while required. Each chamber was also filled with a complete medium, to sustain tissue viability. Adequate pH and oxygenation were ensured by a mixed air/O_2_/CO_2_ gas blown into the device, while the temperature was maintained constant to 37°C by a heating block (Figure 1A).

To evaluate the viability of tissues cultivated ex vivo, an MTT assay was carried out on colon maintained 5 h in the GEVS, and compared with freshly explanted tissues. Data reported in Figure 1B clearly show that tissue overall viability was not affected by the use of GEVS, as no significant differences have been observed with respect to controls.

To check the ability of the system to efficiently and promptly respond to exogenous stimuli, we exposed colon tissues to the well-known endoplasmic reticulum stress inducer Thapsigargin (TG, 5 μg/mL). Therefore, as shown in Figure 1C, we observed no significant differences in tissue overall viability of treated (TG) vs. untreated (CTRL) colon. Then, we evaluated the induction of ER stress, upon TG treatment. To this aim, we compared the expression of specific ER stress markers such as ATF4, ATF6, and spliced XBP1 (XBP1s) [9], between TG treated (TG) vs. untreated (CTRL) samples. Data reported in Figure 1D–F show that the expression levels (mRNA) of all selected UPR markers was increased in tissues treated with TG, compared to matched controls. 

### 3.2. DNBS Induces Tissue Stress and Compromises the Permeability Barrier of Colon Cultivated in a GEVS 

To stimulate the colon tissue inflammation associated with IBD, we decided to expose these organs to a range of concentrations of DNBS (Dinitrobenzene sulfonic acid), a well-known chemical compound used in vivo to stimulate gut inflammation in mice models of IBD [11]. To this aim, tissue perfusing medium was supplemented with 0.5–1.5–2.5 mg/mL of DNBS, or 5 μg/mL of TG, as a control, which did not alter the tissue overall viability (Figure 1G). Then, tissues were homogenized and total RNA or proteins were extracted. The expression of the stress-related factor transglutaminase 2 (TG2) was then evaluated by both qPCR and western blotting analysis, showing a dose-dependent enhanced expression (Figure 1H,I) [12,13].

Gut barrier permeability is controlled by epithelial tight junctions (TJ), multiprotein complexes able to seal together adjacent cell membranes. Altered expression of TJ proteins results in dysregulated tissue permeability, a condition associated with IBD development and progression, which further contributes to tissue inflammation [14,15].

Therefore, we also evaluated the expression of typical TJ proteins, such as Occludin, Claudin-2, and Claudin-15, at the mRNA level [10]. These data are shown in Figure 1J,K,L respectively, and collectively confirmed a dysregulated tissue barrier due to DNBS treatment, as evidenced by a prompt down-regulation of Occludin, while both Claudin-2 and Claudin-15 showing a dose-dependent up-regulation.

### 3.3. DNBS Induces ER Stress in Colon Cultivated in a GEVS

Dysregulation of the endoplasmic reticulum homeostasis, named ER Stress, is emerging as an important pathway associate with IBD development and progression, potentially implicated in tissue inflammation, in both intestinal and colonic mucosa [16,17,18]. Particularly, the effect of ER stress on the pathogenesis of IBD is mainly mediated by impairing the mucosal barrier function, regulating innate or adaptive immune response of the host cells, and modulating the intestinal microbiota [16,17,18].

Moreover, oral administration of Tauroursodeoxycholic acid (TUDCA) and 4-phenylburyrate (4PBA) compounds (two chemical chaperones) consistently alleviated DSS-induced inflammation inhibiting ER stress signaling in gut epithelial cells [19].

**Figure 2:**
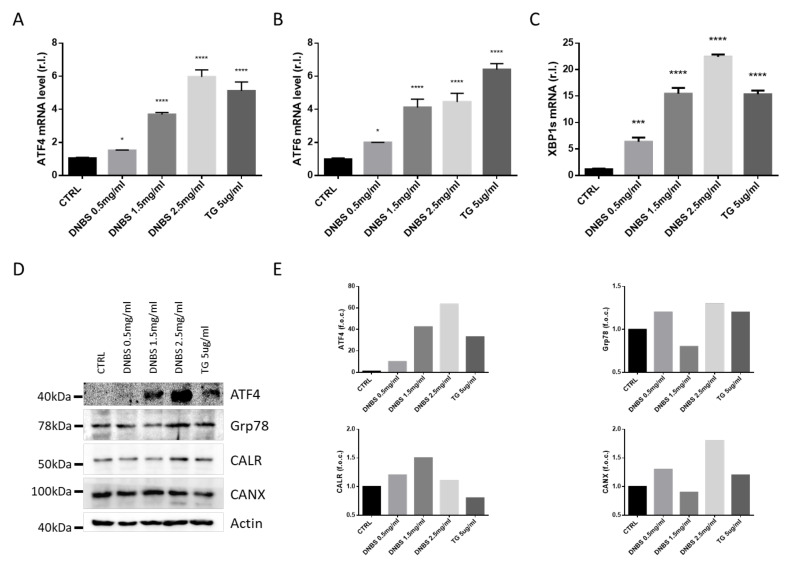
DNBS-induced ER Stress. Tissues were cultivated 5 h in a GEVS and untreated (CTRL) or treated with the indicated concentrations of DNBS, or Thapsigargin (TG), and ATF4 (**A**), ATF6 (**B**), or spliced XBP1 (XBP1s; (**C**)) mRNA levels were evaluated by qPCR. The expression levels of other ER stress markers such as ATF4, Grp78, Calreticulin (CANR), or Calnexin (CALX) were evaluated in the same experimental conditions, by western blotting analysis (**D**). (**E**) Densitometric analysis of western blots reported in D. Data is shown as fold change over control (f.o.c.). Each qPCR panel is representative of experiments performed in triplicate. WBs are representative of three independent experiments. Histograms represent mean ± SD of triplicate sample; **** *p* < 0.0001; *** *p* < 0.001; * *p* < 0.05; ns = not significant.

Therefore, we evaluated the expression of key ER stress markers, such as ATF4 (Figure 2A,D,E), ATF6 (Figure 2B), spliced XBP1 (XBP1s; Figure 2C), Grp78, Calreticulin (CANR), and Calnexin (CALX; Figure 2D,E) [20,21], in colon tissues cultivated 5h in our GEVS, in presence or absence of DNBS, using TG as a positive control [21]. To this aim, the mRNA or protein levels of these markers were evaluated by qPCR or western blotting analysis, and data reported in Figure 2 clearly show a DNBS-dependent upregulation of all the unfolded protein response (UPR) markers.

### 3.4. The Expression of Cytokines Associated to IBD Development Are Modulated by DNBS in Tissues Cultivated in a GEVS

Cytokines are well-known key molecules involved in intercellular communication. Those involved in inflammation are known as pro- or anti- inflammatory cytokines. Although several different cytokines are involved in tissue inflammation, driving immune cells recruitment, differentiation and activation, some of them have been associated with IBD, such as TNFα, IFNγ, IL1β, IL6, IL10, IL15, and IL17 [22,23].

**Figure 3:**
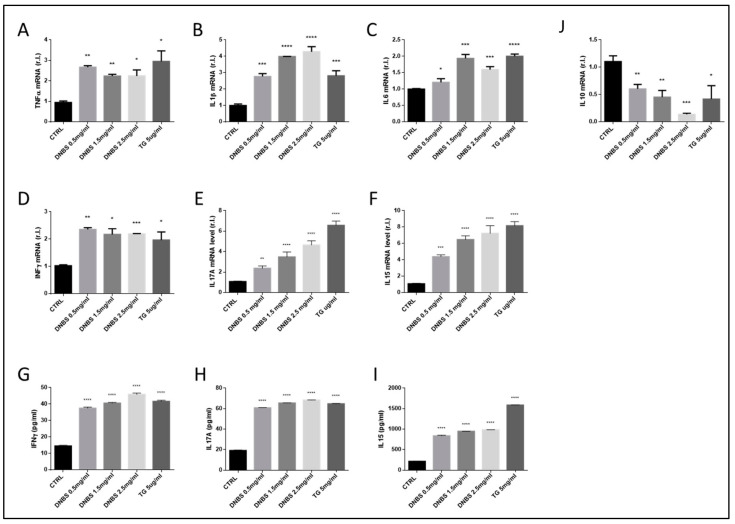
DNBS-modulated IBD-related cytokines expression. Colon tissues were cultivated 5 h as in Figure 2 and the expression of the pro-inflammatory cytokines TNFα (**A**), IL-1β (**B**), IL-6 (**C**), IFNγ (**D**), IL17A (**E**), and IL15 (**F**) was evaluated at mRNA level, by qPCR. The production of IFNγ (**G**), IL17A (**H**), and IL15 (**I**) were also evaluated at the protein level, by ELISA. The expression of the anti-inflammatory cytokine IL10 was also evaluated in the same experimental conditions, by qPCR (**J**). Each panel is representative of experiments performed in triplicate. Histograms represent mean ± SD of triplicate sample; **** *p* < 0.0001; *** *p* < 0.001; ** *p* < 0.01; * *p* < 0.05.

To evaluate the ability of DNBS to stimulate the expression of both pro- and anti- inflammatory cytokines in the colon after 5 h of treatment (in a GEVS), we measured the mRNA and protein levels of these factors, by qPCR and ELISA (TG was used as a control). Data indicate both an enhanced expression of pro-inflammatory cytokines and downregulation of anti-inflammatory cytokines (Figure 3). Indeed, we observed a prompt upregulation of TNFα, IL1β, IFNγ, IL15, and IL17A, starting from the lowest DNBS concentration (0.5 mg/mL), while a consistent upregulation of IL6 expression was evident from 1.5 mg/mL DNBS, at least at mRNA levels. Furthermore, these data were confirmed by an analysis of the protein levels of IFNγ, IL15, and IL17A, by ELISA, which demonstrate a consistent production/release of these cytokines starting from the lowest DNBS concentration (Figure 3G–I). Finally, these results were in line with the down-regulation of the anti-inflammatory IL10 (Figure 3J).

Interestingly, our data also indicate the up-regulation of pro-inflammatory cytokines and the down-regulation of the anti-inflammatory IL10 also in tissues treated with TG (Figure 3).

### 3.5. Colon Tissue Damage, upon Stimulation with DNBS

Chronic inflammation associated with IBD results in epithelial erosion due to cell death induction, immune cell infiltration, edema of the submucosa, and fibrosis.

**Figure 4:**
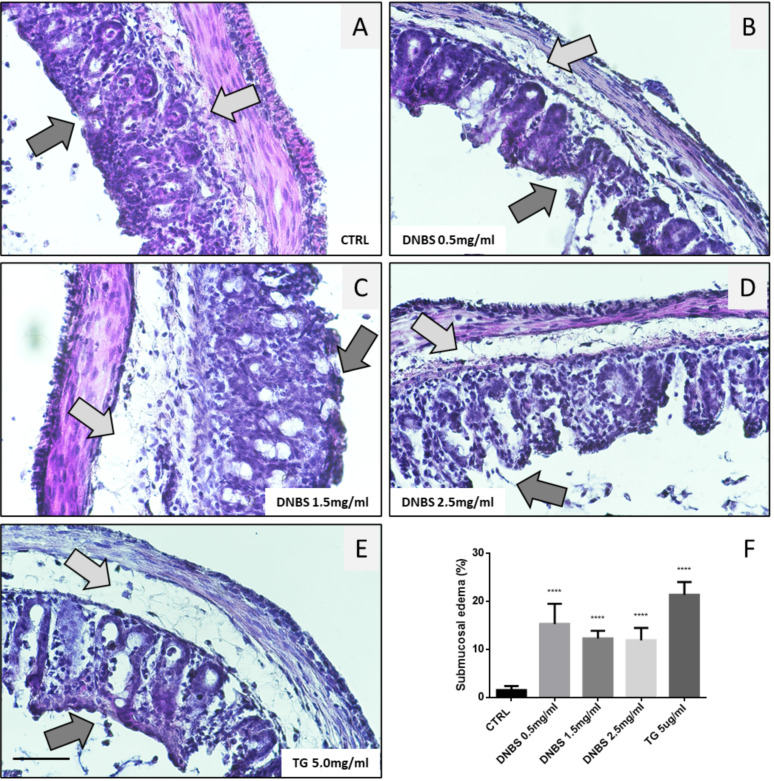
Altered morphology of colon treated with DNBS. Tissues (colon) were cultivated 5 h in the GEVS in absence (CTRL; **A**) or presence of DNBS 0.5 (**B**), 1.5 (**C**), 2.5 mg/mL (**D**), or with TG 5 μg/mL (**E**), and tissue morphology was evaluated in sections stained with H&E (magnification = 40×; bar = 100 μm). Dark and light grey arrows indicate the luminal epithelia and the submucosa, respectively. (**F**) Submucosal edema was evaluated as a percentage of edema thickness relative to the thickness of the colon section. Histograms represent mean ± SD of about 10 measurements/experimental condition; **** *p* < 0.0001.

**Figure 5:**
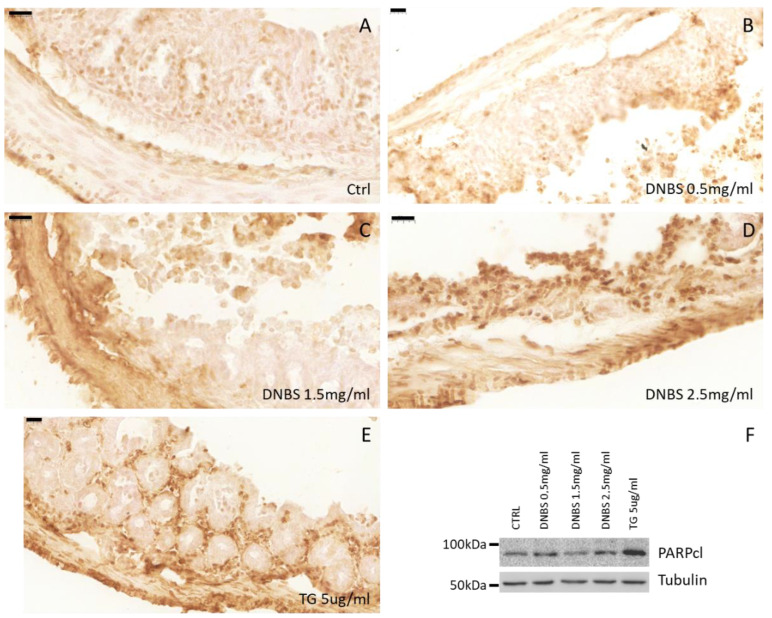
Tissue damage. Tissues (colon) were cultivated 5 h in the GEVS in the absence (CTRL; **A**) or presence of DNBS 0.5 (**B**), 1.5 (**C**), 2.5 mg/mL (**D**), or with TG 5 μg/mL (**E**), and cell death was evaluated by TUNEL assay. (**F**) Part of the tissues were homogenized and total proteins were separated by SDS-PAGE. The membrane was incubated with anti-cleaved PARP. Tubulin was used as a loading control.

Eventually, this condition can also proceed to malignant transformation. To check whether the DNBS-dependent induction of ER stress and pro-inflammatory cytokines production, in our experimental conditions (GEVS) was also paralleled by tissue damage, we evaluated the morphology of colon tissues exposed 5 h to TG or increased concentration of DNBS. As reported in Figure 4, Hematoxylin/Eosin staining of these tissues showed a DNBS-dependent erosion of the luminal epithelial component (dark gray arrows) with a concomitant appearance of submucosal edema (light gray arrows) [24]. The thickness of submucosal edema was measured and reported as a percentage of the total tissue section (Figure 4F). Interestingly, our data indicate a similar trend also in tissue treated with TG (Figure 5E,F).

Finally, to better evaluate the tissue damages resulting from treatments, we also performed a TUNEL assay on tissue sections from colon exposed to DNBS or TG. Data reported in Figure 5 clearly indicate an increase in TUNEL positive cells in tissue exposed to DNBS or TG. These data confirmed the induction of DNBS/TG-induced cell death revealed by western blotting analysis of cleaved PARP, performed on whole tissue lysates, reported in Figure 5F and Supplementary S1.

## 4. Discussion

IBD is an idiopathic, complex, and multifactorial systemic disease of the gastrointestinal tract, characterized by a chronic inflammatory process, thus resulting in tissue damage and, occasionally, in cancer development [25]. Although its precise etiology is still unclear, it is now widely evident that the environment, genetic, microbiota, and immune components are all variably implicated in the onset and progression of the two main forms of IBD, Ulcerative Colitis (UC) and Chron’s Disease (CD) [4]. Therefore, due to the high complexity and heterogeneity of the disease, the development of models to study its pathogenesis and to test new therapeutic approaches are urgently needed. In this context, patients’ biopsies might represent the optimal method, and several protocols have been developed in recent years [26,27,28]. The main limitations of these protocols are represented by invasiveness, limited time of culture, limited sample size, high heterogeneity of the samples and among samples, and loss of both tissue integrity and microenvironment. On the other hand, in vitro approaches based on epithelial cell cultures in both 2D and 3D conditions (spheroids/organoids) cannot represent ‘translational’ models, compared to both biopsies and animal models [29,30,31]. Indeed, these models can be efficiently used to study intracellular signaling pathways, specific cell damages, and response to treatments but, similarly to biopsies, the loss of microenvironment represents the main limitation of these approaches.

Animal models indubitably represent the main preclinical approach used to study IBD development, progression and test new potential therapeutic regimens. Several protocols have been developed, with DSS, DNBS, or TNBS representing the main chemical compounds used in these models. Although with differences, TNBS is particularly indicated to mimic Chron’s Disease [32,33], while both DSS and DNBS are prone to induce Ulcerative Colitis [34,35,36,37]. However, all these models are differently characterized by drug-dependent inflammation of the gastrointestinal tract, pro-inflammatory cytokines production/release, immune cell infiltration, submucosal edema, and luminal epithelial death/erosion [33]. Although highly heterogeneous and patient dependent, altogether they all represent markers of human IBD. However, working with animals is expensive, time-consuming, and involves important animal suffering. Here we have provided an ex vivo colon tissue culture (Gut-Ex-Vivo System, GEVS) which although cannot completely replace animal models, can however be used to study IBD development at the molecular level, allowing the analysis of the whole gut tissue. Indeed, the system can be loaded with colon freshly explanted which are viable till the end of the experiments, and UC can be induced by 5 h exposure to DNBS. This approach can be compared to acute UC and is characterized by a prompt ER stress induction as evidenced by upregulation of typical UPR markers such as ATF4, ATF6, and XBP1s. This is not surprising since ER stress is emerging as a key signaling pathway involved in IBD pathogenesis and tissue dysfunction, driving the onset of inflammation [16,38,39]. It is also important to note that these results support the reliability of our model since a DNBS-mediated ER stress induction has also been described in an animal model of IBD [40]. Interestingly, the upregulation of these markers seems to be dose-dependent, potentially indicating increased tissue damage related to drug concentration, as also indicated by the dose-dependent upregulation of the stress protein TG2 [12].

A defective mucosal barrier, resulting in compromised permeability, is another all-maker of IBD, resulting in the exposition to luminal content and triggers an immunological response, thus mediating inflammation [41]. Barrier dysfunction is associated with impaired mucus production and abnormal expression and/or relocation of specific molecules, such as the tight junction proteins [41]. The key role played by these components is sustained by clinical evidence showing as an anti-TNFα therapy reduces mucosal inflammation, thus restoring physiological barrier functions, and TJ protein expression [42]. Consistent with these notions, we observed a DNBS-induced altered expression of TJ proteins such as Occludin, Claudin-2, and Claudin-15, indicating a dysregulated tissue barrier.

IBD-related gut inflammation and tissue dysfunction/damage are closely related to immune response, in which cytokines play a key role, and which concentration represents a valuable tool to evaluate disease severity [43]. Interestingly, we observed a dose-dependent increased expression of a panel of pro-inflammatory cytokines such as TNFα, IFNγ, IL1β, IL6, IL15, and IL17A. In parallel, a decreased expression of the anti-inflammatory IL10, was also found. Collectively, these data further sustain the validity of our model, in mimicking the human disease. 

Noteworthy, both TJ protein expression deregulation and cytokines expression observed in DNBS-treated tissues were also evident in colon exposed to TG, further sustaining the key role played by ER stress in driving IBD. Further studies are required to characterize the link among UPR, cytokines, and TJ protein expression in IBD, at a molecular level.

Acute/chronic inflammation also drives gut epithelium damage and altered tissue morphology in patients affected by IBD [44]. This consists of luminal epithelium erosion, mainly due to cell death induction, cryptitis, submucosal edema, fibrosis, and altered tissue thickness. The variability of these symptoms and their severity are closely related to the chronicity of the inflammatory event, which could eventually lead to a malignant transformation. Due to the short-term insult in our GEVS (5 h), we did not expect a massive tissue alteration. In fact, a shortening of the colon length and a thickening of the tissue observed in mouse models chronically exposed to DNBS [45] were not evident even at the maximum drug concentration used in this study.

However, a change in tissue morphology was evident in colon exposed to DNBS and mainly characterized by the appearance of submucosal edema and luminal epithelium erosion. The overall tissue damage is paralleled by an increased cell death induction, contributing to epithelial erosion, as evidenced by both TUNEL assay and the cleavage of the caspase target PARP.

On note, a consistent shortening of the colon was evidenced in a longer experiment, in which the colon was exposed for 18 h to 2.5 mg/mL of DNBS, by using our GEVS (data not shown).

## 5. Conclusions

The development of appropriate models used to study human diseases is crucial for analyzing their pathogenesis and development at the molecular level and for testing potential therapeutic approaches. Diverse and heterogeneous models are available today to study most human diseases ranging from 2D cell culture systems, 3D models, biopsies, organ-on-chip, to animal models. Undoubtedly, all of these models have positive aspects but also several limitations. Therefore, we believe that using a combination of models is the best option.

Here we have described a novel model based on dynamic organ culture, a Gut-Ex-Vivo (GEVS) system, to study the pathogenesis of IBD-related intestinal inflammation. We have demonstrated that our model is consistent, reliable, effective, flexible, cost-effective, and time-efficient. It is also important to point out that the system can also potentially be used to test new therapeutic approaches, such as new compounds, probiotics, prebiotics, and nutraceuticals.

## Data Availability

Data is contained within the article or supplementary material.

## Supplementary Materials

The following are available online at https://www.mdpi.com/article/10.3390/biology10070605/s1, Table S1: Primer’s sequence; Supplementary S1: Tissue damage.
