# Supplementary material for: A Gut-Ex-Vivo System to Study Gut Inflammation Associated to Inflammatory Bowel Disease (IBD)
Source: Biology (Basel). 2021 Jun 30;10(7):605. doi: 10.3390/biology10070605 (PMC8301106; doi:10.3390/biology10070605)
Supplement: Supplementary file 1 [file biology-10-00605-s001.zip › Suppl_S1.pdf]

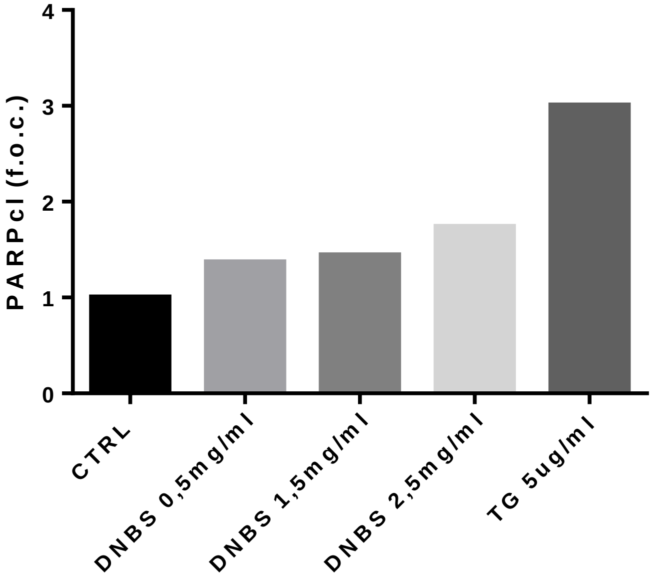

Supplementary S1. Tissue damage. Densitometric analysis of cleaved PARP (PARPcl) western blotting reported in Figure 5F. PARPcl expression was normalized using Tubulin as loading control. Data are reported as fold over control (f.o.c.).
