# Supplementary material for: A Gut-Ex-Vivo System to Study Gut Inflammation Associated to Inflammatory Bowel Disease (IBD)
Source: Biology (Basel). 2021 Jun 30;10(7):605. doi: 10.3390/biology10070605 (PMC8301106; doi:10.3390/biology10070605)
Supplement: Supplementary file 1 [file biology-10-00605-s001.zip › Supplementary Table1.pdf]

Supplementary Table1. Primers sequence.

Primer	Sequence
TNFα_F	CCTCCCTCTCATCAGTTCTAT
TNFα_R	ACTTGGTGGTTTGCTACG
IL10_F	TGAATTCCCTGGGTGAGA
IL10_R	CCACTGCCTTGCTCTTATT
IL1β_F	GGACCCATATGAGCTGAAAG
IL1β_R	TGTCGTTGCTTGGTTCTC
IL6_F	GAGGAGACTTCACAGAGGATA
IL6_R	ATTTCCACGATTTCCCAGAG
IL15_F	CAGCAAGGACCATGAAGA
IL15_R	GGCTGAGTTCCACATCTAAC
IL17A_F	CGCAATGAAGACCCTGATAG
IL17A_R	CTTGCTGGATGAGAACAGAA
INFγ_F	CCACATCTATGCCACTTGAG
INFγ_R	CTCTTCCTCATGGCTGTTTC
ATF4_F	GTTTAGAGCTAGGCAGTGAAG
ATF4_R	CCTTTACACATGGAGGGATTAG
ATF6_F	GATGGTGACAACCAGAAAGA
ATF6_R	TGGAGGTGGAGGCATATAA
XBP1s_F	AGTCCGCAGCAGGTG
XBP1s_R	GGTCCAACTTGTCCAGAATG
TG2_F	AAGAGCGAAGGGACATACT
TG2_R	TGAGCACAGACCCATCTT
CLD15_F	GGGACCCTCCACATACTT
CLD15_R	CATACTTGGTTCCAGCATACA
CLD2_F	CCTCGCTGGCTTGTATTATC
CLD2_R	AAAGACTCCACCCACTACA
OCLN_F	TCTTTGGAGGAAGCCTAAAC
OCLN_R	CTGCTCTTGGGTCTGTATATC
GAPDH_F	TTCAACGGCACAGTCAAG
GAPDH_R	CCAGTAGACTCCACGACATA

***Note**: XBP1s_F/R = primers designed to amplify the spliced (mature) mRNA of XBP1.*
